# A rare presentation of an ACTH‐producing high‐grade large cell neuroendocrine carcinoma with Cushing’s syndrome

**DOI:** 10.1002/ccr3.5168

**Published:** 2021-12-13

**Authors:** Francis Essien, Christine Persaud, David Dado, Rina Eden, Joshua Tate, George Shahin

**Affiliations:** ^1^ Department of Internal Medicine David Grant Medical Center Travis Air Force Base Fairfield California USA; ^2^ Division of Nephrology Department of Internal Medicine Keesler Medical Center Keesler Air Force Base Biloxi MS USA; ^3^ Division of Pathology Department of Internal Medicine Keesler Medical Center Keesler Air Force Base Biloxi MS USA; ^4^ Division of Endocrinology Department of Internal Medicine Keesler Medical Center Keesler Air Force Base Biloxi MS USA; ^5^ Division of Hematology/Oncology Department of Internal Medicine Keesler Medical Center Keesler Air Force Base Biloxi MS USA

**Keywords:** chemotherapy, Cushing's syndrome, high‐grade neuroendocrine tumors (HGNET), large cell type, pancreatic neuroendocrine tumors (P‐NETs), poor differentiation

## Abstract

High‐grade neuroendocrine tumors (HGNET) are rare neoplasms composed of neural and hormonal with only around 42 cases reported in the last 20 years1. Herein, we describe a rare case of pancreatic HGNET, large cell type, associated with a Cushing's syndrome presentation.

## Graphical abstract

An ACTH‐producing FDG avid pancreatic high‐grade neuroendocrine tumor with diffuse metastasis on PET/CT presenting as Cushing's syndrome treated with cytotoxic chemotherapy.

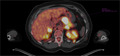

## 1 INTRODUCTION

Neuroendocrine tumors (NET) are a highly aggressive heterogeneous group of neoplasms that arise from a combination of neural and hormonal cell types. The most common tumors arise from the lungs, small intestine, appendix, and pancreas; less frequently from the thyroid, parathyroid, pituitary, and adrenal glands.^1^ There are several different classifications based on the differentiation of tumor cells compared to neighboring non‐neoplastic cells. However, in general tumors that are well differentiated are low grade, and those with poor differentiation are high grade. Large cell neuroendocrine carcinoma was first proposed by Travis et al. in 1991 and subsequently classified as HGNET by the World Health Organization (WHO) International Histological Classification of Tumours.^1^ HGNET is a rare tumor with unclear clinicopathologic features and poor mortality given the high rate of metastasis.^1^ Herein, we describe a rare case of pancreatic HGNET, a large cell type, associated with a Cushing's syndrome presentation. Additionally, due to lack of prospective data in the literature regarding treatment of large cell neuroendocrine tumor, there is a reliance on case reports to develop treatment protocols.

## 2 CASE REPORT

**FIGURE 1:**
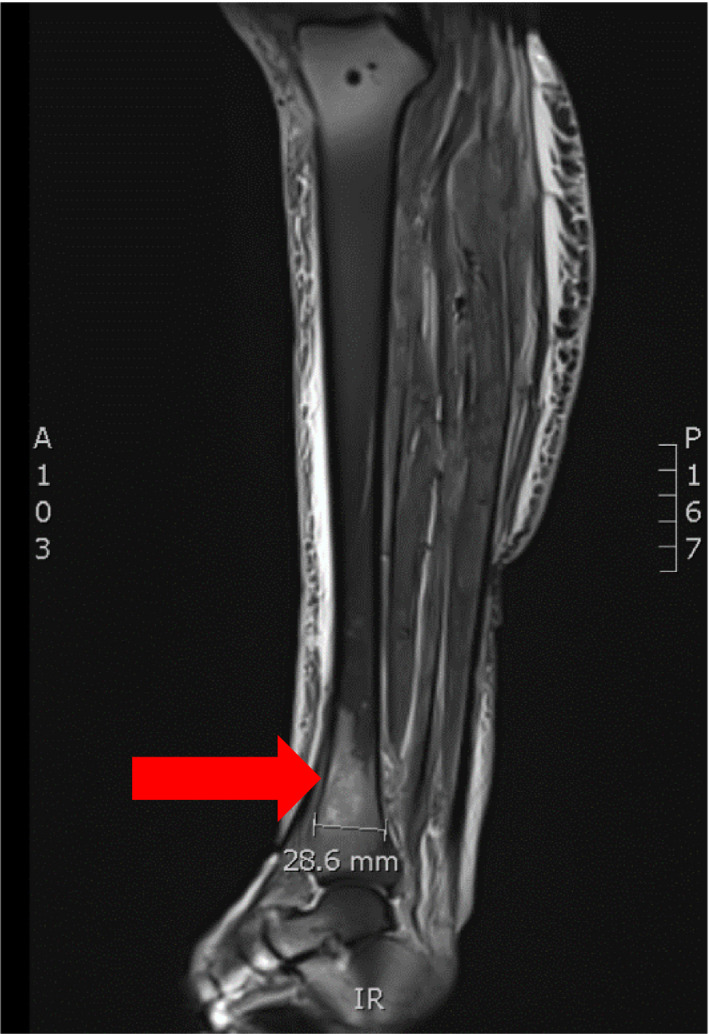
MRI right tibia‐fibula, sagittal image, showing lesion within the distal tibia with areas of nodular abnormal signal noted throughout the remainder of the tibia

**FIGURE 2:**
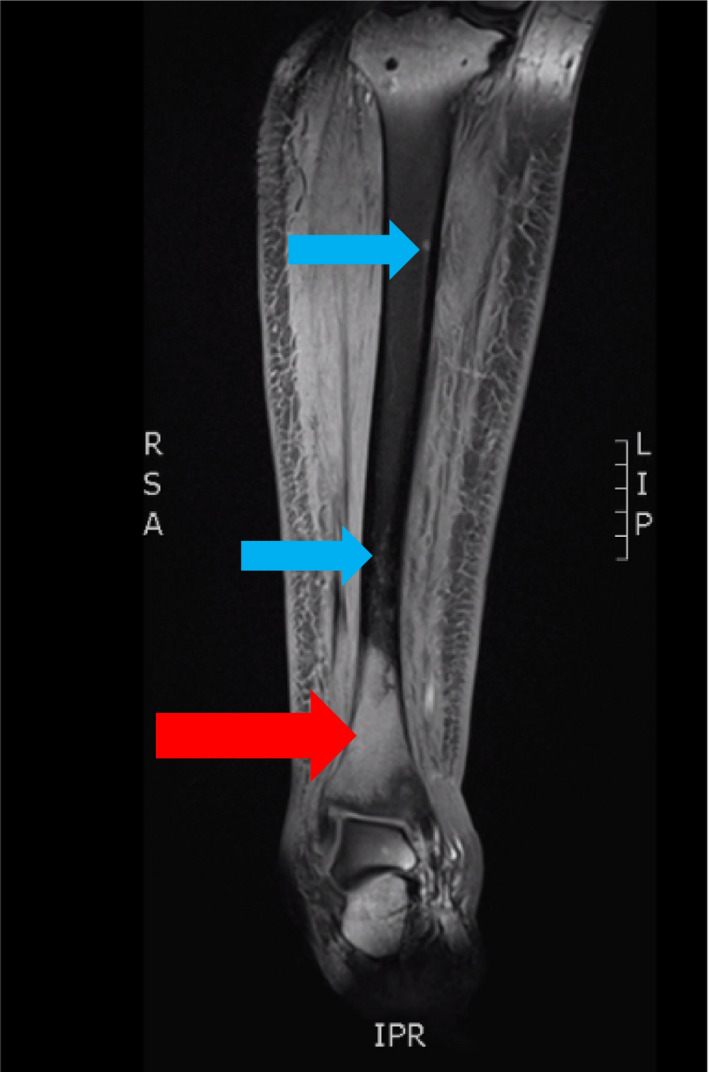
MRI right tibia‐fibula, coronal image, showing infiltrative enhancing lesion involving the distal tibia with satellite lesions suggested throughout the remainder of the tibia and central portion of the talus

**FIGURE 3:**
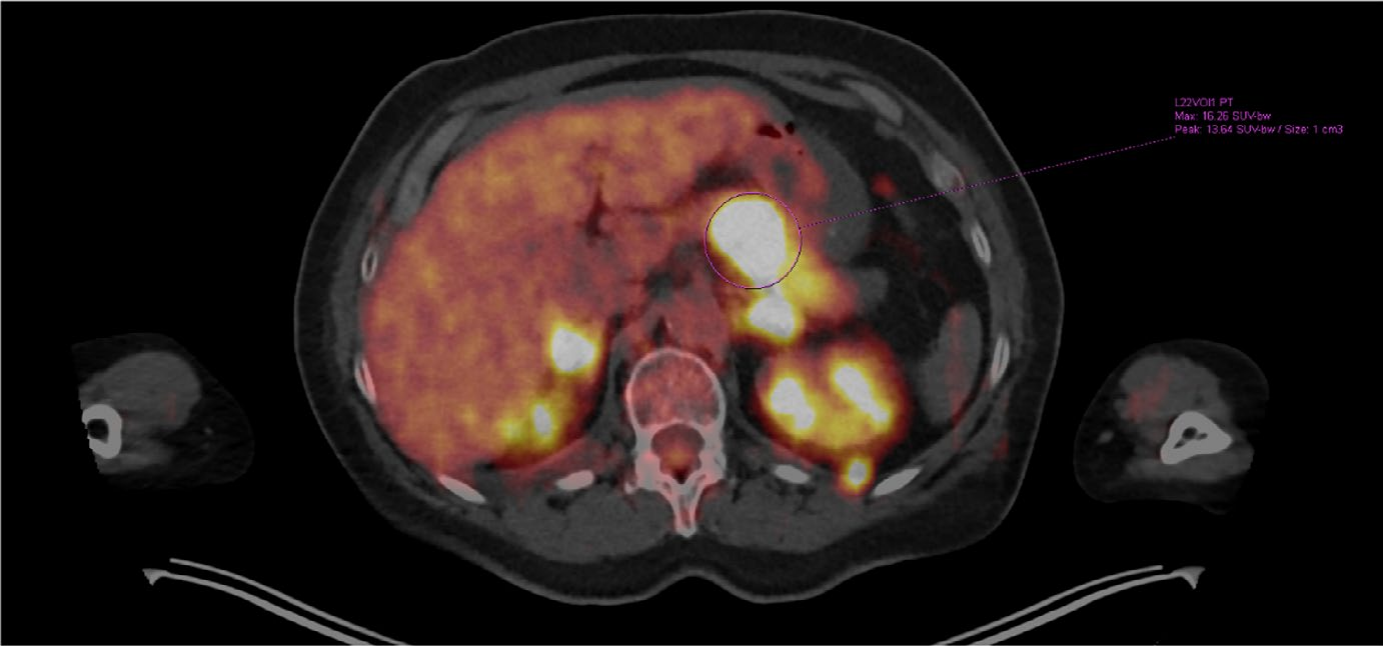
PET/CT whole body survey, transverse image, showing increased FDG avidity of adrenal glands, numerous metabolically active renal lesions, and FDG avid mass within the body of the pancreas

**FIGURE 4:**
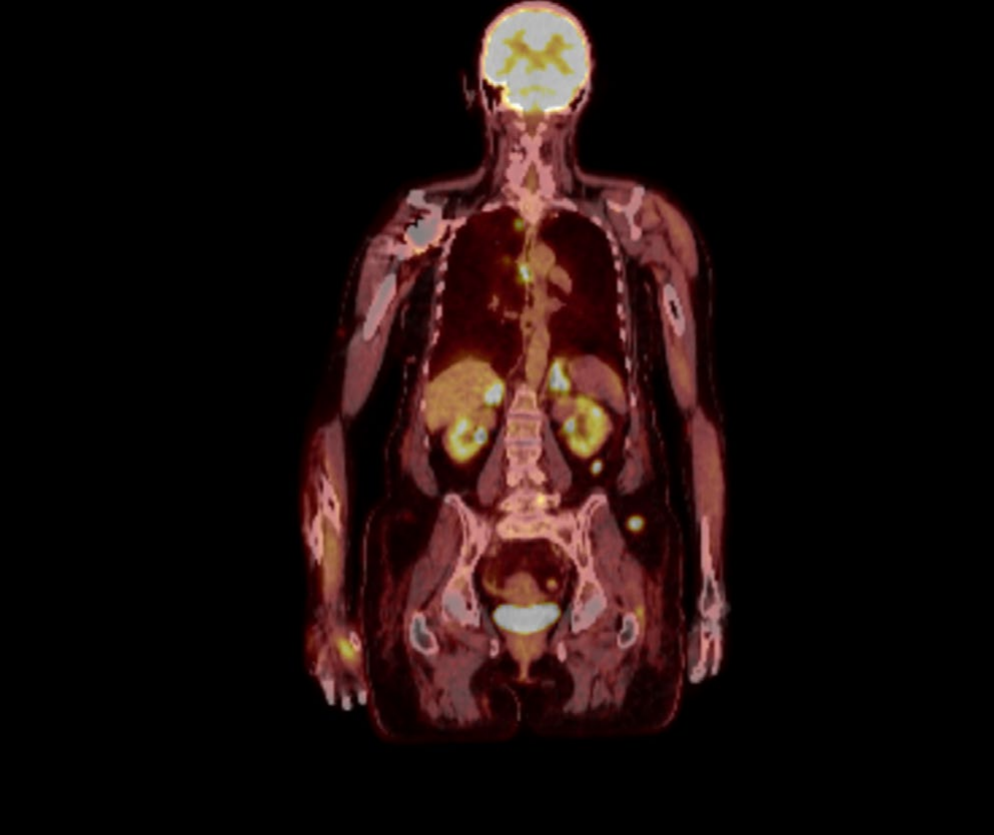
PET/CT whole body survey, coronal image, showing diffuse metastatic disease, scattered FDG avid osseous lesions throughout the axial and appendicular skeleton, supraclaviclar/mediatinal lymphadenopathy, increased FDG avidity of adrenal glands with a left adrenal mass, numerous metabolically active renal lesions, and numerous metabolically active soft tissue densities scattered throughout subcutaneous fat

A 61‐year‐old female patient presented to her primary care physician in late December 2020 with increasing right leg/ankle pain unresponsive to conservative therapy, elevated blood pressure, and concerns of new hirsutism. Subsequent MRI of her right leg revealed an infiltrative enhancing lesion of the distal tibia concerning metastatic foci (Figures 1 and 2). Follow‐up PET/CT demonstrated diffuse metastatic disease (Figures 3 and 4). An MRI brain was notable for diffuse boney metastasis without overt evidence of intraparenchymal disease. Initially, this was thought to be an adrenal gland primary tumor due to noted hirsutism and elevate cortisol; however, a full hormonal assessment was completed (Table 1). Ultimately, the results revealed both an elevated cortisol and elevated ACTH, favoring a non‐adrenal and suspected pancreatic origin HGNEC (fluorodeoxyglucose [FDG] avid mass seen on PET), given the association of ACTH‐producing NET arising from islet tumor cells.

**TABLE 1:** Laboratory data workup

Test name	Result value and units	Reference range
Renin	<0.167 (L) ng/ml/h	(0.167–5.380)
Aldosterone	<1.0 ng/dl	(0.0–30.0)
Norepinephrine	371 pg/ml	(0–874)
Epinephrine	<15 pg/ml	(0–62)
Dopamine	80 (H) pg/ml	(0–48)
Normetanephrine	37.1 pg/ml	(0.0–191.8)
Metanephrine	<10.0 pg/ml	(0.0–88.0)
Vanillylmandelate	3.0 mg/L	Undefined
Vanillylmandelate	5.8 mg/24 h	(0.0–7.5)
Epinephrine	4 mcg/L	Undefined
Epinephrine	8 mcg/24 h	(0–20)
Norepinephrine	19 mcg/L	Undefined
Norepinephrine	37 mcg/24 h	(0–135)
Dopamine	78 mcg/L	Undefined
Dopamine	151 mcg/24 r	(0–510)
Cortisol	65.52 (H) mcg/dl	(5.27–22.45)
Adrenocorticotrophic hormone	487.0 (H) pg/ml	(7.2–63.3)
Cortisol free 24 h urine	>1000 mcg/L	Undefined
Cortisol 24 h urine	>1940 (H) mcg/24 h	(6–42)
Aldosterone 24 h urine	<2.50 mcg/L	Not Established
Aldosterone 24 h urine	<4.85 mcg/24 h	(0.00–19.00)
Normetanephrine 24 h urine	112 mcg/L	Undefined
Normetanephrine 24 h urine	217 mcg/24 h	(131–612)
Metanephrine 24 h urine	27 mcg/L	Undefined
Metanephrines 24 h urine	52 mcg/24 hr	(36–209)
Potassium 24 h urine volume	1940 mL	(800–1800)
Potassium 24 h urine	57.9 mmol/L	Not Established
Potassium 24 h urine	112.326 mmol/24 h	(25–125)
Creatinine [Mass/volume] 24 h urine	50.60 mg/dl	(28–259)
Dehydroepiandrosterone sulfate	294.0 (H)	(18.9–205.0)
Androstenedione	2220 (H) ng/dl	(17–99)
Testosterone	97 (H) ng/dl	(<8–53)
17‐Hydroxyprogesterone	275 ng/dl	(Follicular 15–70) (Luteal 35–290)
Estradiol	21.1 pg/ml	(11–52.5)

**FIGURE 5:**
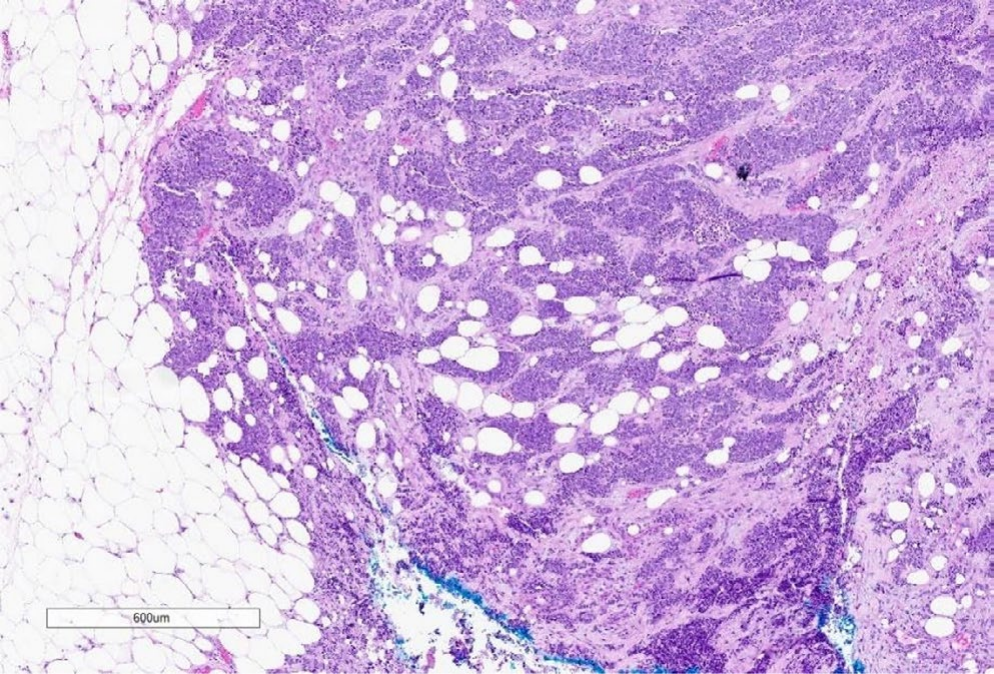
Hemotoxylin and eosin stain at 40×. Nests of malignant neuroendocrine cells are seen infiltrating into the subcutaneous fat

**FIGURE 6:**
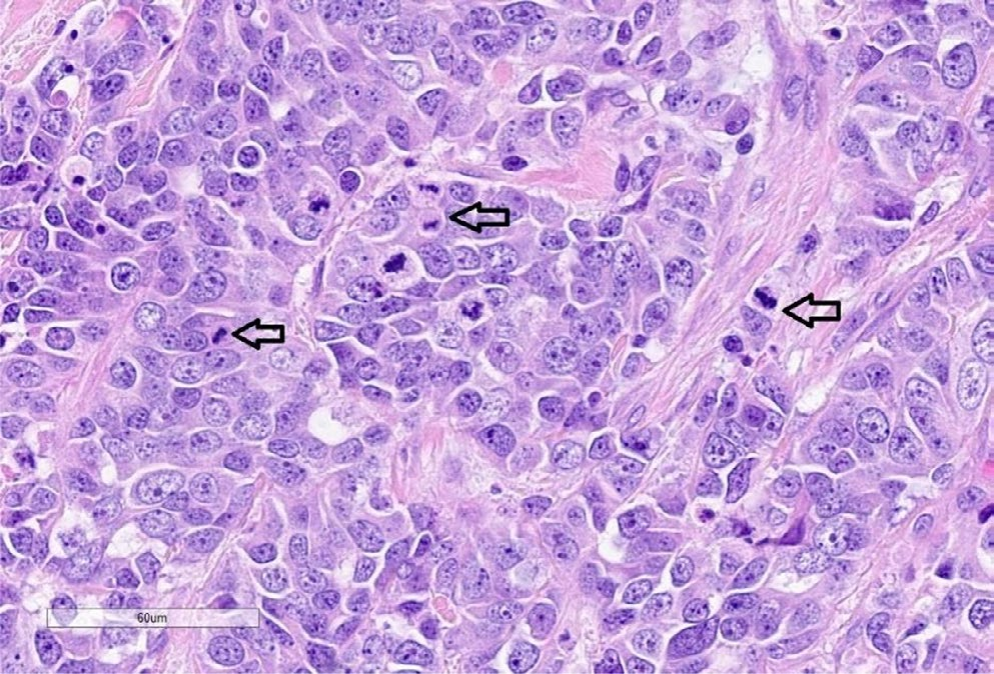
Hemotoxylin and eosin stain at 400×. Malignant cells show classic neuroendocrine “salt‐and‐pepper” chromatin pattern within nuclei. Frequent mitoses are seen (arrows)

In February 2021, the patient had a biopsy of a subcutaneous breast lesion with initial pathology demonstrating malignant infiltrative proliferation within soft tissues that features pleomorphic nests of cells, nucleoli with salt‐and‐pepper chromatin, higher nuclear: cytoplasmic ratios, and greater than 40 mitoses per 2 mm E^E2 (Figures 5 and 6). Immunohistochemical stains were performed and positive for Lu‐5, CK‐7 (patchy), TTF‐1, chromogranin, and synaptophysin, and negative for CK20, SOX10, GATA‐3, and p40. These findings were consistent with HGNET.

Due to the aggressive nature and high burden of her disease, the patient was admitted to the hospital upon receipt of her pathology results. Hematology/oncology and endocrinology were consulted. She was initiated on cytotoxic chemotherapy with carboplatin and etoposide. The patient was also initiated on ketoconazole to inhibit steroidogenesis in the setting of ectopic ACTH production/Cushing's syndrome. The patient tolerated chemotherapy administration well without evidence of tumor lysis syndrome. On Day 5, however, she developed an ileus requiring the placement of a nasogastric tube. Laboratories demonstrated severe cytopenias with absolute neutrophil count (ANC) <600. On Day 7, she began to experience respiratory distress with imaging notable for diffuse bilateral pulmonary opacities with a pleural effusion. The patient was intubated due to impending respiratory failure. Bronchoscopy and thoracentesis were performed at that time. She was initiated on empiric antimicrobials, daily filgastrim, and stress dose hydrocortisone given concern for relative adrenal insufficiency. Infectious workup including blood cultures, urine cultures, cerebral spinal fluid studies, and BAL returned negative. Unfortunately, the patient continued to deteriorate with the development of multi‐organ failure and decreasing neurologic response despite sedation holds. In March 2021, the patient was transitioned to comfort care, terminally extubated, and passed shortly thereafter.

## 3 DISCUSSION

Pancreatic neuroendocrine tumors (P‐NETs) are rare, with ectopic (ACTH) secretion syndrome an even rarer clinical manifestation with only a few cases reported in the literature. The most common ectopic ACTH‐producing tumors are thoracic (bronchial and thymic) and gastroenteropancreatic (NET), followed by medullary thyroid carcinoma, small cell lung cancer, and pheochromocytoma.^1^ These tumors are clinically distinct from the more common, well differentiated, and low‐ or intermediate‐grade neuroendocrine tumors. There are limited cases of HGNET diagnosed and reported in the literature; thus, there is not standardized staging, classifications, or treatment regimens at this time.

The incidence of P‐NETs is challenging to categorize due to the fact that most international cancer registries do not collect information on tumor grade, but available data suggest that high‐grade neoplasms are rare. Registries from the Netherlands, the United States, and Norway suggest an annual incidence between 0.2 and 0.5 per 100,000 inhabitants.^1^, ^2^ Within the last few decades, the incidence is noted to be increasing, but this may coincide with the changes in the nomenclature, and classification, resulting in more awareness. Unlike its lung cohort, the risk factors for P‐NETs are not well elucidated, and more data are being compiled. The largest case series reported a medium overall survival (OS) of 13.2 months and a 3 year OS of 8.7% for all patients.^1^, ^2^ Histopathologically, HGNET has an architecture consistent with neuroendocrine differentiation. The cells are arranged in an organoid, trabecular or palisading pattern, with prominent necrosis. Evidence of neuroendocrine differentiation is demonstrated by immunoreactivity for chromogranin and synaptophysin.^3^ The mitotic rate tends to be more extensive larger than what is seen in atypical carcinoid cells with WHO criteria stating cutoff above >10/2 mm^2^.^3^ Our patient had a mitoses rate greater than 40 mitoses per 2 mm E^E.

These tumors have an aggressive natural history constituted by early, rapid, widespread metastasis. HGNETs are very difficult malignancies to diagnose due to the subtle presentation.^1^ Ectopic adrenocorticotropic hormone (ACTH) secretion syndrome is a rare clinical manifestation but is responsible for 15% of all cases of Cushing's syndrome.^4^, ^5^ This has been previously well documented in the subset of small cell carcinoma of the lung, but not the pancreatic subset. However, pancreatic islet tumors constitute 1 of the 4 histological subsets (small cell, pheochromocytoma, and carcinoid tumors) of ectopic ACTH‐producing tumors, and therefore, this possibility can be extrapolated to the neuroendocrine tumor.^2^ Most patients present with a number of cushingoid features including facial plethora, ecchymoses, muscle weakness, hypertension, and laboratory derangements such as severe hypokalemia and glucose intolerance. Our patient presented with clinical features of hirsutism, virilization, hypokalemia, and metabolic alkalosis.

The treatment for metastatic P‐NETs is difficult to determine due to the general lack of data from prospective trials. Some patients present with potentially resectable metastatic disease and hence may benefit from a combination of chemotherapy and surgical resection. For patients who do not meet these criteria, such as our patient, recommendations are to treat in similar fashion to its cohort, small cell carcinoma. Chemotherapy regimens used in this setting are platinum‐based (cisplatin or carboplatin) with etoposide.^2^, ^3^ This regimen has been well established in several retrospective studies as a first‐line treatment. Recently, there has been experimentation with several classes of drugs to include rapamycin (mTOR) inhibitors such as everolimus, temozolomide, and capecitabine.^2^ Various treatments are being explored with an aim for DNA synthesis interruption by alkylation, pyrimidine analogs, or platinum exposure. Recently, there has been exploration into molecularly therapies with sunitinib and PD‐1 inhibitor Pembrolizumab. Preliminary data suggest that HGNETs are less responsive but if there are high levels of mutations or microsatellite instability, immunotherapies such as pembrolizumab should be considered early in the course of the disease.^6^ Due to mineralocorticoid excess and severe electrolyte/metabolic derangements, with elevated cardiac markers, carboplatin was used in place of cisplatin to reduce complications.

Hormonal hypersecretion plays a large role in tumor morbidity and mortality depending on the type of hormone. Cushing's syndrome results from excess cortisol release, which can be caused by a number of etiologies to include ectopic ACTH production, as seen in our patient. This can result in a number of systemic issues to include hypertension, myopathy, and osteoporosis, poor wound healing, and psychiatric disturbance.^4^ The serum ACTH and cortisol were both very elevated in our patient. In order to control the excessive amount of tumor‐driven hormone release, there are three classes of drugs available for use to include steroidogenesis inhibitors, neuromodulators of ACTH release, and glucocorticoid receptor‐blocking agents.^5^ Current guidelines suggest steroidogenesis inhibitor mitotane as the first line in combination with chemotherapy.^7^ Due to limitations in the Tricare network, many steroidogenesis inhibitors and/or glucocorticoids inhibitors are not readily available including, mitotane, metyrapone, and mifepristone. Ketoconazole, used in our patient, works through the inhibition of 17–20 desmolase, blockade of 17‐hydroxylase, and inhibition of 21‐ and/or 11‐hydroxylase.^8^ Time to peak concentration is around 2 h^9^; therefore, even though it might take several weeks to achieve full inhibition, Ketoconazole should have a readily available effect.

This case highlights several important clinical implications. First, HGNET is a rare neoplasm that is often difficult to differentiate. Treatment, while available, is limited and associated with poor outcomes. Second, treatment should consist of a multidisciplinary discussion in order to facilitate an effective plan of action for patients with this disease process. Third, although mitotane is the first line of adrenolytic therapy, not every facility may have this readily available.^10^ The past decade has seen a shift of focus from empiric therapeutic trials to pathological and molecular profiling‐based studies that may help define and select patient subtypes that could benefits from subtype‐specific treatment.

## ACKNOWLEDGEMENTS

None.

## CONFLICT OF INRERESTS

None.

## AUTHOR CONTRIBUTIONS

The authors confirm contribution to the paper as follows: Study conception and design: Francis Essien D.O., David Dado D.O., Rina Eden D.O., Joshua Tate M.D., George Shahin M.D.; Data collection: Francis Essien D.O., Rina Eden D.O., George Shahin M.D.; Analysis and interpretation of results: Francis Essien D.O., Joshua Tate M.D., Rina Eden D.O., George Shahin M.D.; Draft manuscript preparation: Francis Essien D.O., Christine Persaud D.O.; All authors discussed the results and contributed to the final manuscript.

## CONSENT

Written informed consent was obtained from the patient to publish this report in accordance with the journal's patient consent policy.

## Data Availability

Data sharing is not applicable to this article as no new data were created or analyzed in this study.
